# European Consensus on the Management of Sensitized Kidney Transplant Recipients: A Delphi Study

**DOI:** 10.3389/ti.2024.12475

**Published:** 2024-04-11

**Authors:** Lucrezia Furian, Oriol Bestard, Klemens Budde, Emanuele Cozzi, Fritz Diekmann, Nizam Mamode, Maarten Naesens, Liset H. M. Pengel, Soren Schwartz Sorensen, Fabio Vistoli, Olivier Thaunat

**Affiliations:** ^1^ Kidney and Pancreas Transplantation Unit, Department of Surgical, Oncological and Gastroenterological Sciences, School of Medicine and Surgery, University of Padua, Padua, Italy; ^2^ Kidney Transplant Unit, Vall d’Hebron University Hospital, Barcelona, Spain; ^3^ Department of Nephrology and Medical Intensive Care, Charité University Medicine Berlin, Berlin, Germany; ^4^ Transplant Immunology Unit, Department of Cardiac, Thoracic and Vascular Sciences, School of Medicine and Surgery, University of Padua, Padua, Italy; ^5^ Experimental Nephrology and Transplant Laboratory, August Pi i Sunyer Biomedical Research Institute (IDIBAPS), Barcelona, Spain; ^6^ King’s College London, London, United Kingdom; ^7^ Department of Microbiology, Immunology and Transplantation, Faculty of Medicine, KU Leuven, Leuven, Belgium; ^8^ Erasmus MC Transplant Institute, Erasmus University Medical Center Rotterdam, Rotterdam, Netherlands; ^9^ Department of Neurology, Rigshospitalet, Copenhagen University Hospital, University of Copenhagen, Copenhagen, Denmark; ^10^ University of Pisa, Pisa, Italy; ^11^ Department of Biothecnological and Applied Clinical Sciences, University of L’Aquila, L’Aquila, Italy; ^12^ Service de Transplantation, Néphrologie et Immunologie Clinique, Hospices Civils de Lyon, Lyon, France

**Keywords:** kidney transplantation, desensitization, immunomodulation, systematic review, Delphi

## Abstract

An increasing number of sensitized patients awaiting transplantation face limited options, leading to fatalities during dialysis and higher costs. The absence of established evidence highlights the need for collaborative consensus. Donor-specific antibodies (DSA)-triggered antibody-mediated rejection (AMR) significantly contributes to kidney graft failure, especially in sensitized patients. The European Society for Organ Transplantation (ESOT) launched the ENGAGE initiative, categorizing sensitized candidates by AMR risk to improve patient care. A systematic review assessed induction and maintenance regimens as well as antibody removal strategies, with statements subjected to the Delphi methodology. A Likert-scale survey was distributed to 53 European experts (Nephrologists, Transplant surgeons and Immunologists) with experience in kidney transplant recipient care. A rate ≥75% with the same answer was considered consensus. Consensus was achieved in 95.3% of statements. While most recommendations aligned, two statements related to complement inhibitors for AMR prophylaxis lacked consensus. The ENGAGE consensus presents contemporary recommendations for desensitization and immunomodulation strategies, grounded in predefined risk categories. The adoption of tailored, patient-specific measures is anticipated to streamline the care of sensitized recipients undergoing renal allografts. While this approach holds the promise of enhancing transplant accessibility and fostering long-term success in transplantation outcomes, its efficacy will need to be assessed through dedicated studies.

## Introduction

The incidence of chronic kidney disease is rocketing worldwide and it is widely acknowledged that kidney transplantation represents the best therapeutic option for patients reaching end-stage kidney failure [1]. However, there is a rapid increasing number of highly sensitized patients waitlisted worldwide, who have limited access to transplantation. 2024 OPTN data from US [2] show that 11% of waiting list kidney transplant candidates can be defined as highly sensitized (HS), displaying a cPRA>80% (5% of listed patients display >98% cPRA), and 45% of candidates have some degree of sensitization with a cPRA>1%. The Eurotransplant data report that 35% of candidates display a virtual PRA>0% in 2023 [3] and the percentage of >85% PRA listed patients increased from 3.4% in 2014 to 6% in 2019, whereas the percentage of sensitized patients at any degree (PRA between 6% and 84%) remained stable (14%). Country-specific reports show a percentage of HS candidates varying from 20% to 30% depending on the assay utilized (20% with cPRA>98% in Spain, 25% with cPRA >85% in France, 28% with cRF >85% in United Kingdom). In Australia the proportion appears similar, with 30% patients having cPRA >80% [4–9]. In the absence of consensual evidence-based data regarding the way these high immunological risk patients should be managed, a large proportion of them remain on chronic dialysis, which detrimentally impact both on their quantity and quality of life and represents a higher financial burden for the society [10–13].

**FIGURE 1:**
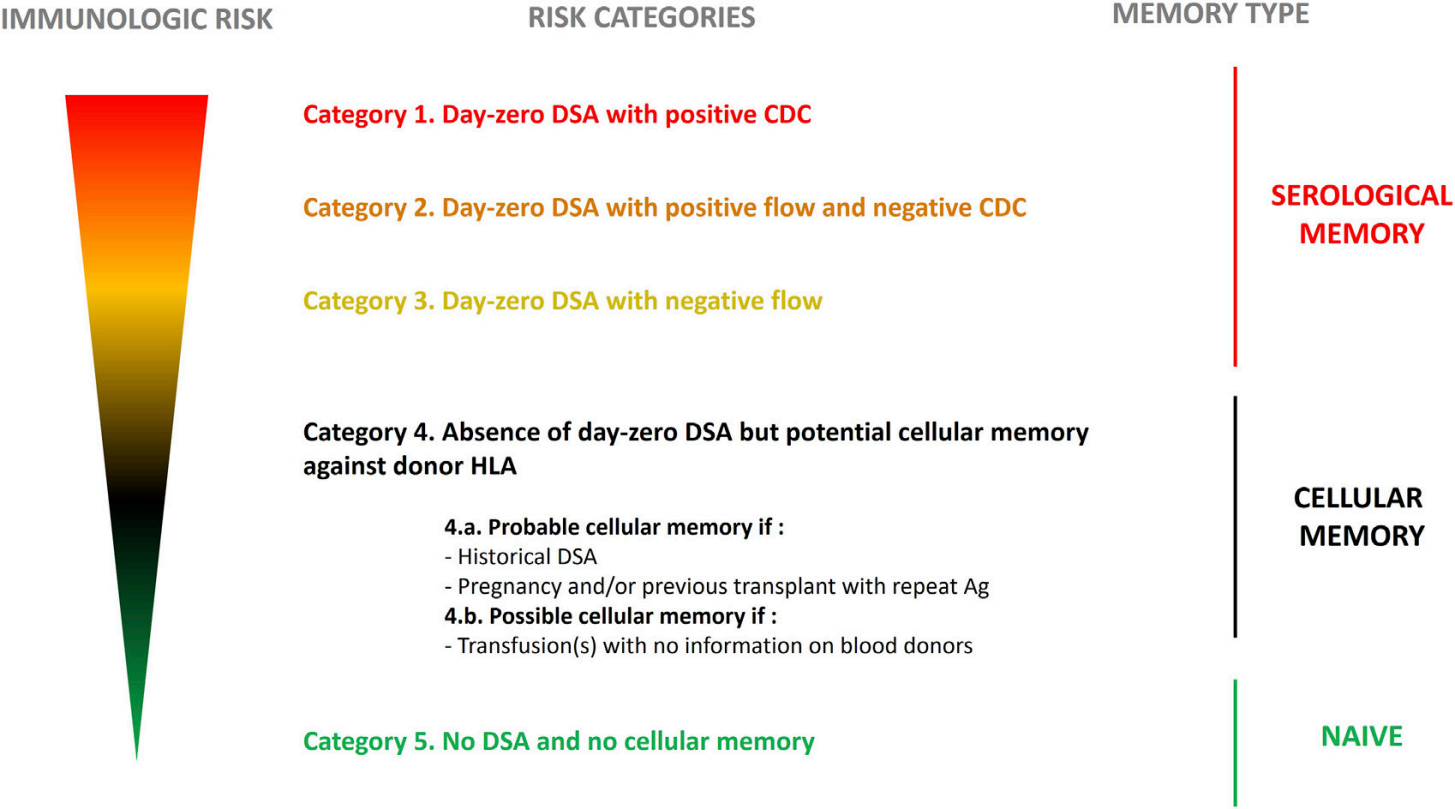
Stratification for risk in kidney transplant recipients based on level of sensitization. Stratification was proposed by the ENGAGE working group 2021. Edited with permission.

In 2021, the European Society for Organ Transplantation (ESOT) initiated the EuropeaN Guidelines for the mAnagement of Graft rEcipients (ENGAGE) program. That same year the ENGAGE working group proposed a stratification of the humoral risk for candidate to a solid organ transplantation [1]. Based on patient’s “immunological” history and the results of single-antigen bead assay, cytotoxic (CDC) and flow cytometry crossmatches, sensitized candidates can be distributed into five categories (Figure 1) with decreasing risk for AMR from Category 1 (Patients with day 0 DSA with positive CDC crossmatch) to 5 (patients with no DSA and no cellular memory).

Following the publication of this stratification, the ENGAGE II working group was established to discuss how patient management should be adapted in the five ENGAGE categories. The ENGAGE II working group includes members from across Europe, selected among ESOT/EKITA recognised experts in the field of transplant immunology, kidney transplantation, and the management of high-risk kidney transplant candidates or recipients. The approach was based on two consecutive steps. In the first step, a systematic review of the literature was conducted leading to the generation of a list of evidence-based proposals on induction therapies, antibody removal strategies and new biological drugs and maintenance immunosuppression. Consensus about these proposals was then established in each ENGAGE category using the Delphi methodology.

## Methods

The Steering Committee of ENGAGE II working group included members of the ENGAGE I and the TLJ WS06, all the previous experts accepted either as panellists or scientific committee. The requisites to be involved were to be representative of different European Countries with experience in desensitization based on scientific publications or participation to multicentre clinical studies on HS patients. None of the contacted centres or experts declined to participate, witnessing the high interest of this topic in selected transplant centres. The Scientific Committee for the evidence-based evaluation and consensus generation consisted of ten members, Lucrezia Furian (Italy; co-Chair), Olivier Thaunat (France; co-Chair), Nizam Mamode (United Kingdom), Oriol Bestard (Spain), Maarten Naesens (Belgium), Klemens Budde (Germany), Fabio Vistoli (Italy), Emanuele Cozzi (Italy), Soren Schwartz Sorensen (Denmark) and Fritz Diekmann (Spain), all academic kidney transplant experts.

### Systematic Literature Review

A systematic search of the published literature was conducted to identify studies reporting on induction regimens in sensitised kidney transplant recipients and studies reporting antibody removal strategies and new biological agents in low to very high-risk kidney transplant recipients (Supplementary Figure S1).

Two clinical questions were formulated according to the PICO (Population, Intervention, Comparison, Outcome) structure to define the search strategy as well as the inclusion and exclusion criteria for selection of publications. Scientific committee met several times online to define the scope, the PICOs, and discuss the results.

The first clinical question was “*What is the efficacy of different induction agents or protocols on transplant outcomes in low to very high-risk kidney transplant recipients*?”. The population (P) was defined as low to very high-risk kidney transplant recipients (all ages), intervention (I) as induction agents or protocols, no comparators (C) were considered and the outcome (O) was defined as 1-year patient and graft survival, acute rejection rates type of rejection according to the Banff Classification, 5- and 10-year graft and patient survival, and development of DSAs. Systematic reviews, randomised controlled trials, registry analyses, case series were considered relevant. Publications were excluded if they were published before 2000 or in any language other than English.

The second clinical question was “*What are the antibody removal strategies and maintenance immunosuppression available to facilitate the access to kidney transplantation and to obtain acceptable outcomes in sensitized recipients*?”. For this question the P was defined as adult sensitized patients, the I as antibody removal strategies and maintenance immunosuppression, no C was considered and the O was defined as AMR, infections, graft function, graft survival, patient survival. Systematic reviews randomised controlled trials, registry analyses, case series were considered relevant. Publications were excluded if they were published before 1995 or in any language other than English. The decision to exclude publications prior to 2000 and 1995, respectively, was taken because more recent publications generally included advancements in antibody detection technologies, diagnostic criteria for rejection and immunosuppressive treatment changes, among others. However, older papers that included high-quality research could be included as supporting evidence, with expert group agreement.

### Literature Search Strategy, Study Selection and Data Collection

**FIGURE 2:**
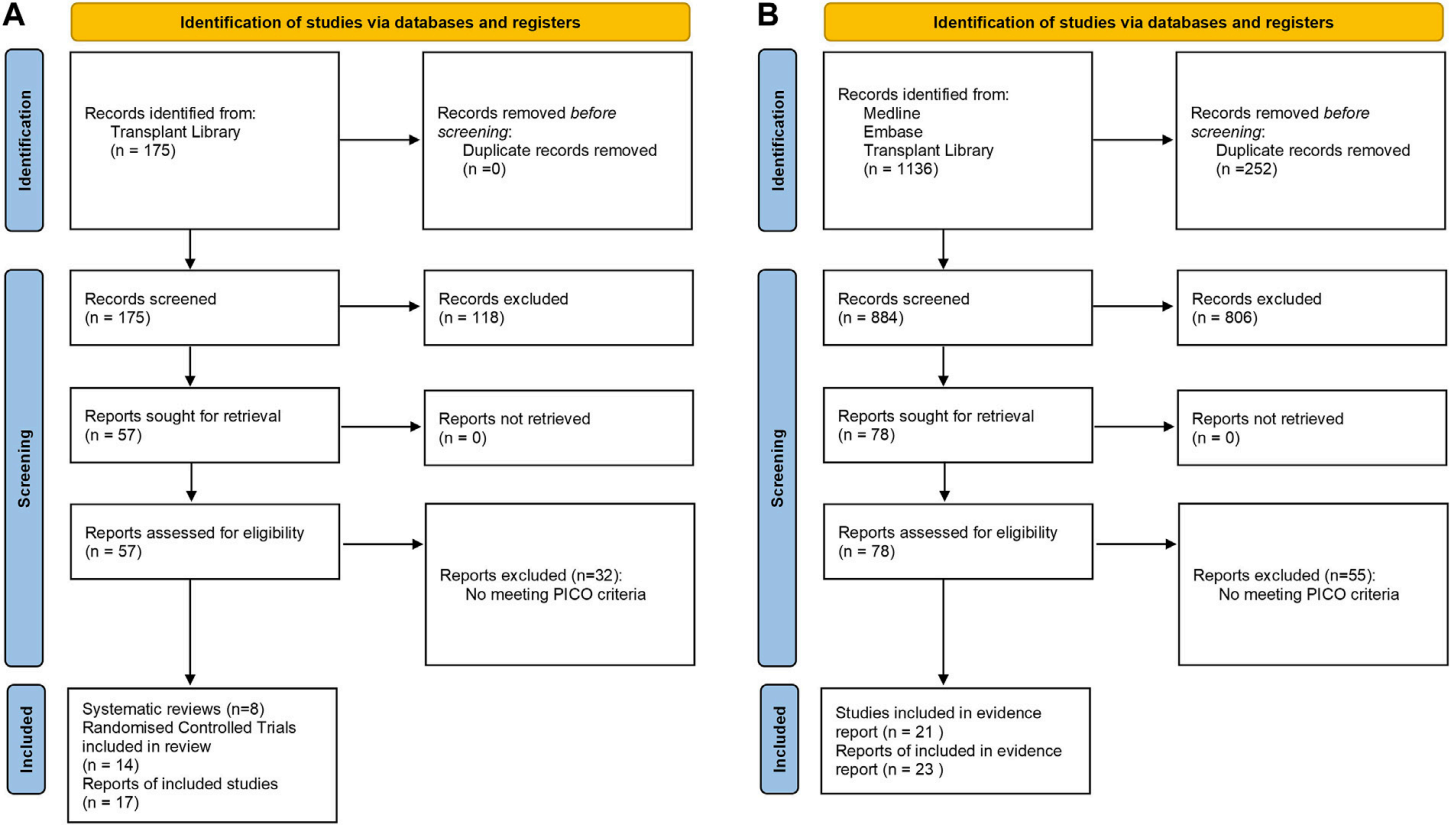
PRISMA flow diagrams for **(A)** efficacy of induction agent and **(B)** antibody removal strategy.

Literature searches for both clinical questions were developed by the Centre for Evidence in Transplantation, University of Oxford, United Kingdom. The search strategy including the list of search queries used per each bibliographic source is provided in Supplementary Table S1. The literature searches were conducted in the Transplant Library (www.transplantlibrary.com), Medline^®^ and Embase^®^ databases, and included free text and controlled vocabulary terms. The titles and abstracts were screened by one reviewer and a list of potentially eligible reports was identified. The review of the literature was refined by a subgroup for each PICO, consisting of two members of the scientific committee who independently evaluated the evidences in the literature. Figure 2 describes both PRISMA flow diagram for the study selection process.

### Consensus Statement Development

Based on the evidence generated through the systematic literature search, the clinical members of the induction therapies, antibody removal strategies and maintenance immunosuppression subgroups drafted statements on induction, desensitization and immunomodulation. Statements were developed for ENGAGE risk categories 1–4b. No specific statements were developed for risk category 5 as these transplant candidates, who present with no DSA and no cellular memory that indicates heightened risk of rejection, were not the focus of the current work. Draft statements for the Delphi process were discussed, revised and approved by the full working group. Statements were then presented to a larger number of experts who qualified as voting members of the Delphi panel (Supplementary Figure S1).

### Delphi Methodology

We employed the Delphi methodology to achieve a global view of current desensitization and immunomodulation strategies during kidney transplantation from a clinical immunology perspective. The process was undertaken between May and September 2022. The Delphi Review Group members were selected by the Scientific Committee based on their specialty (nephrologists, transplant surgeons, immunologists) and their experience in the care of kidney transplantation recipients (minimum of 5 years). An online questionnaire was sent in two waves to nephrologists, transplant surgeons and immunologists of the selected countries. For the first wave, panel members were invited to vote individually on whether they agree, partially agree or disagree with each statement. In case of disagreement or partial disagreement, panel members were asked to briefly explain the reason for their disagreement/partial disagreement with the statement and were invited to re-write the statement as they considered more appropriate. Data were analysed globally. The level of agreement or disagreement was defined by the Scientific Committee when 75% of more of the experts agree on the assessment. Following completion of the first wave, those statements for which consensus had not been achieved were re-written and clarified with some definitions by the Scientific Committee according to the insights provided by the panel members for disagreed/partially disagreed. The second wave consisted of the rewritten statements that had not achieved consensus during the first wave.

## Results

For the systematic literature search regarding the first clinical question, a total of 175 publications were identified from Transplant Library. For the second clinical question, 1,136 publications were identified from Medline^®^, Embase^®^ and Transplant Library databases. A total of 43 statements were developed by the Scientific Committee based on the systematic literature review (Supplementary Table S2).

### Delphi Review Group

Considering the highly specific topic addressed by the questionnaire, different strategies of recruitment were simultaneously taken (Supplementary Table S3). The Delphi Review Group consisted of 53 experts from across Europe (Supplementary Table S4).

### Category 1 Patients (DSA Present With Positive CDC Crossmatch at Day 0)

**FIGURE 3:**
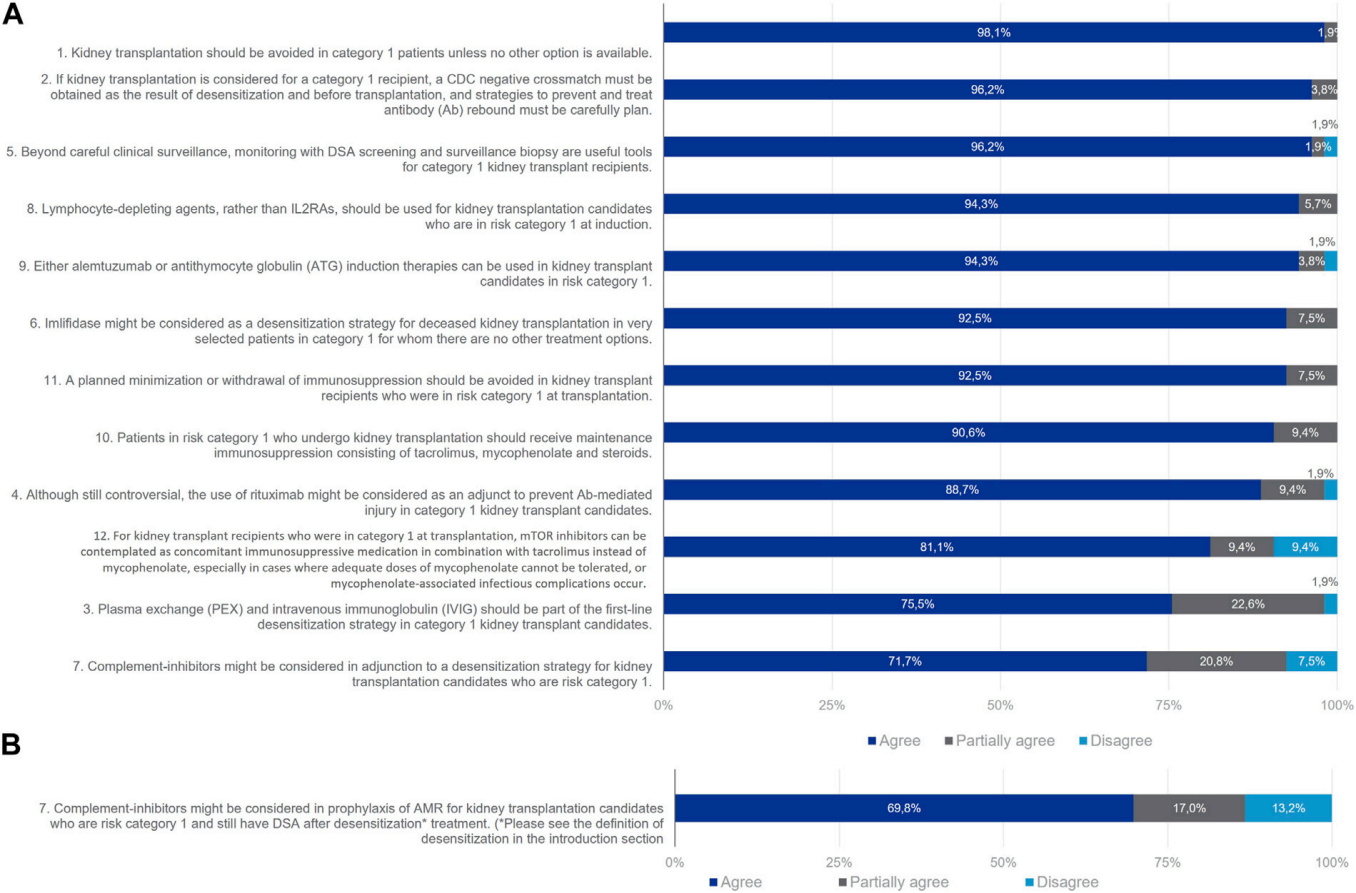
Panellist responses for Category 1 (patients with DSA present and positive CDC crossmatch at D0 and Day 0) after **(A)** Wave 1 and **(B)** Wave 2.

All statements for this group reached consensus except for Statement 7 referring to the use of complement inhibitors as an adjunction to a desensitization strategy (Figure 3). In all, 98% of the Delphi Review Group agreed that kidney transplantation should be avoided unless no other options is available. Most members agreed that if kidney transplantation is considered, a CDC negative crossmatch must be obtained through desensitization before transplantation, and strategies to prevent and treat antibody rebound must be carefully planned (agreement rate 96%). Useful tools beyond careful clinical surveillance are monitoring with DSA screening and surveillance biopsy (agreement rate 96%). There was good agreement that plasma exchange (PEX) and intravenous immunoglobulin (IVIg) should be part of the first line desensitization strategy to provide a negative CDC crossmatch prior to transplantation (agreement rate 75%). Also, imlifidase might be considered as a desensitization strategy for deceased kidney transplantation in very selected patients for whom there are no other treatment options (agreement rate 92%). Regarding induction therapies, experts agreed that T-lymphocyte-depleting agents should be used in these patients rather than IL-2RA (agreement rate 94%). T-cell depleting therapy such as alemtuzumab or antithymocyte globulins (ATG) can be used (agreement rate 94%). The B-cell depleting agent rituximab might be considered as an adjunct to prevent antibody rebound (agreement rate 89%). It was agreed by 91% of the Delphi Review Group that patients in Category 1 should receive maintenance immunosuppression with tacrolimus, mycophenolate and steroids. Also, mTOR inhibitors can be considered in combination with tacrolimus instead of mycophenolate, especially when it cannot be tolerated or when infectious complications due to mycophenolate occur (agreement rate 81%). A total of 92% of the Delphi Review Group agreed that a planned minimization or withdrawal of immunosuppression should be avoided in these patients.

Consensus remained elusive regarding the utilization of complement inhibitors as a prophylactic measure against antibody-mediated rejection (AMR) for patients in this particular cohort who retained donor-specific antibodies (DSA) post-desensitization treatment. In the initial assessment, 72% of the Delphi Review Group supported the notion that complement inhibitors could be considered as an adjunct to desensitization strategies, while 21% expressed partial agreement and 8% dissented. Given the absence of consensus after the first round, the statement underwent refinement for the second round, incorporating a specific definition of desensitization (herein strictly referring to drugs or procedures designed to diminish the titre of anti-donor antibodies, either directly or by targeting antibody-producing cells or their precursors). Nevertheless, consensus remained unattainable in the second wave, with 70% of the Delphi Review Group endorsing the revised statement, 17% offering partial agreement, and 13% dissenting from the statement.

### Category 2 Patients (DSA Present With Positive Flow and Negative CDC Crossmatch at Day 0)

**FIGURE 4:**
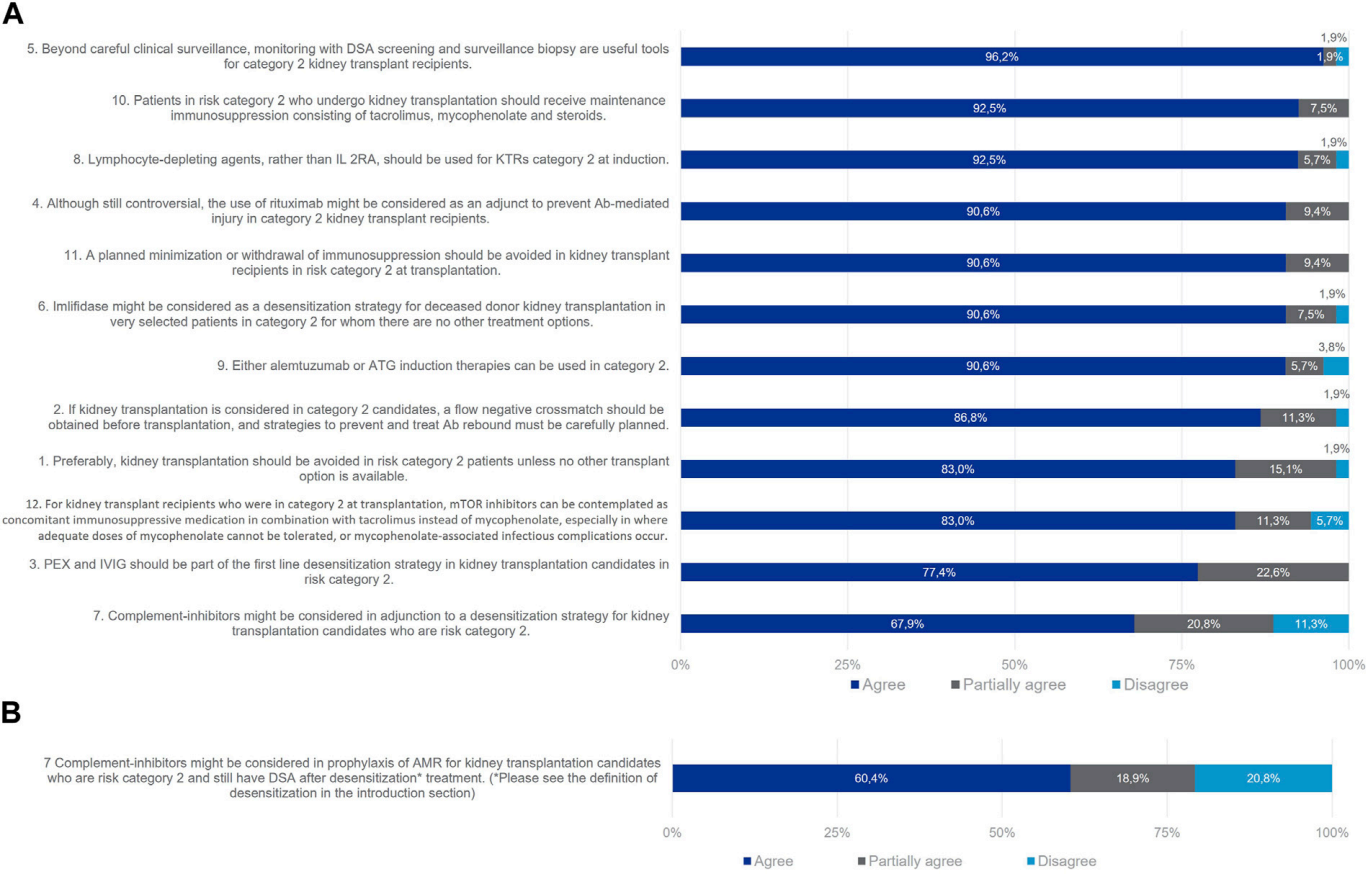
Panellist responses for Category 2 (patients with DSA, negative CDC crossmatch and positive now cytometry crossmatch on Day 0) after **(A)** Wave 1 and **(B)** Wave 2.

For risk Category 2 patients, all statements reached consensus except statement 7 referring to the use of complement inhibitors in prophylaxis of AMR (Figure 4). 83% of the Delphi Review Group agreed that, preferably, kidney transplantation should be avoided, but if there are no other options, it could be considered on a case-by-case basis. In that case, strategies to prevent and treat antibody rebound must be cautiously planned (agreement rate 87%). Useful tools beyond careful clinical surveillance are monitoring with DSA screening and surveillance biopsy (agreement rate 96%). As agreed by 77% of the Delphi Review Group, PEX and IVIg should be part of the first line desensitization strategy, to provide a negative crossmatch prior to transplantation. Also, imlifidase might be considered for deceased kidney transplantation in selected patients for whom there are no other treatment options (agreement rate 91%). The Group agreed that T-lymphocyte-depleting agents should be used as induction therapy in these patients, rather than interleukin 2 receptor antagonists (IL-2RAs; agreement rate 93%). Alemtuzumab or antithymocyte globulins (ATG) can be used (agreement rate 91%). The B-cell depleting agent rituximab might be considered as an adjunct to antibody-mediated injury (agreement rate 91%). Immunosuppression should be maintained for this group, as agreed by 93% of the Delphi Review Group with tacrolimus, mycophenolate and steroids. Also, mTOR inhibitors can be contemplated in combination with tacrolimus instead of mycophenolate, especially when the latter cannot be tolerated or when infectious complications due to mycophenolate occur (agreement rate 83%). Moreover, planned minimizations or withdrawal of immunosuppression should be avoided in these patients (agreement rate 91%).

As for patients of Category 1, consensus proved elusive on the statement concerning the use of complement inhibitors as an adjunct to desensitization strategies for the prophylaxis of AMR in patients retaining donor-specific antibodies (DSA) post-desensitization treatment. In the initial wave, 68% of the Delphi Review Group concurred that complement inhibitors could be considered in tandem with desensitization strategies, 21% partially agreed, and 11% disagreed. In the second wave, the statement was refined to specifically address AMR prophylaxis in patients with persisting DSA. Despite this focus, consensus further diminished for the rephrased statement, with 60% in agreement, 19% partially in agreement, and 21% in disagreement. Overall, while the Delphi results may not endorse the use of complement inhibitors as AMR prophylaxis, there remains interest in exploring this therapeutic class for treating confirmed episodes of AMR.

### Category 3 Patients (DSA Present and Negative Flow and CDC Cross Match at Day 0)

**FIGURE 5:**
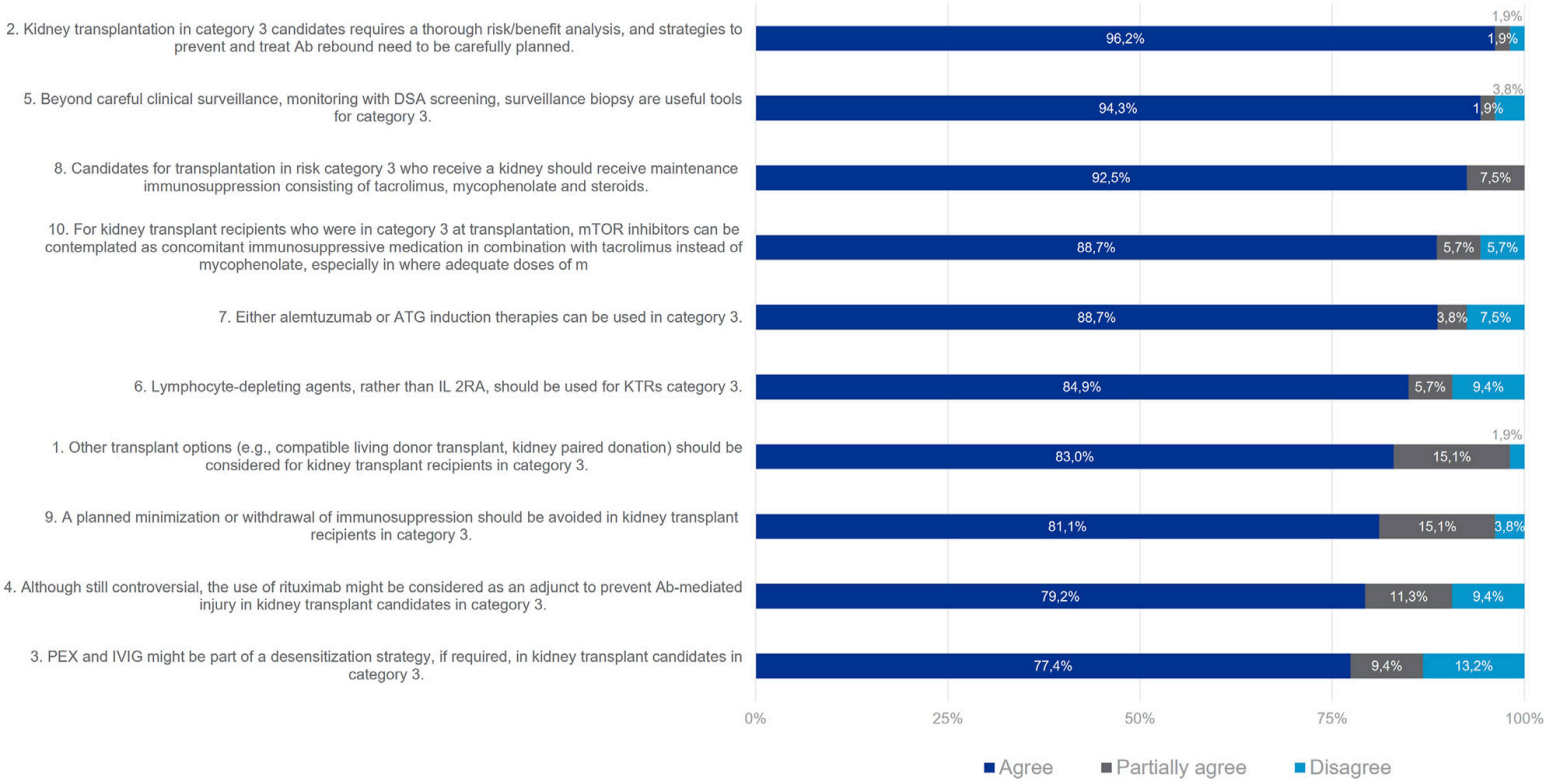
Panellist responses for Category 3 (patients with DSA and negative flow cytometry crossmatch on Day 0).

For Category 3 patients, consensus was reached for all proposed statements at Wave one (Figure 5). 83% of the Delphi Review Group agreed that other options for transplantation (such as compatible living donor transplant or kidney paired donation) should be objectively considered for kidney transplant candidates in Category 3, since these patients are at higher immunological risk than those in categories 4 and 5. Moreover, 96% of the Group agreed that these patients require a thorough risk/benefit analysis, and strategies to prevent and treat antibody rebound need to be carefully planned. Useful tools beyond clinical surveillance are monitoring with DSA screening and surveillance biopsy (agreement rate 94%). For desensitization, PEX and IVIg might be considered (agreement rate 77%). Additionally, rituximab might be considered as an adjunct to prevent antibody-mediated injury (agreement rate 79%). For induction therapy, T-lymphocyte-depleting agents should be used, rather than IL-2RAs (agreement rate 85%). Alemtuzumab or ATG can be used (agreement rate 89%). According to 93% of the Delphi Review Group, immunosuppression should be maintained, with tacrolimus, mycophenolate and steroids. Also, mTOR inhibitors can be contemplated in combination with tacrolimus instead of mycophenolate, especially when the latter cannot be tolerated or when infectious complications due to mycophenolate occur (agreement rate 89%). Further, planned minimizations or withdrawal of immunosuppression should be avoided in these patients (agreement rate 81%).

### Category 4 Patients (Without DSA on Day 0 But With Potential Cellular Memory Against Donor HLA)

In ENGAGE stratification [1], category 4 was further divided into category 4a, with “probable” cellular memory, in case of positive history of DSA, pregnancy and/or previous transplant with repeated antigens, and category 4b with “possible” cellular memory if they have a history of transfusions and/or pregnancies with no information on the HLA type patient was exposed to.

**FIGURE 6:**
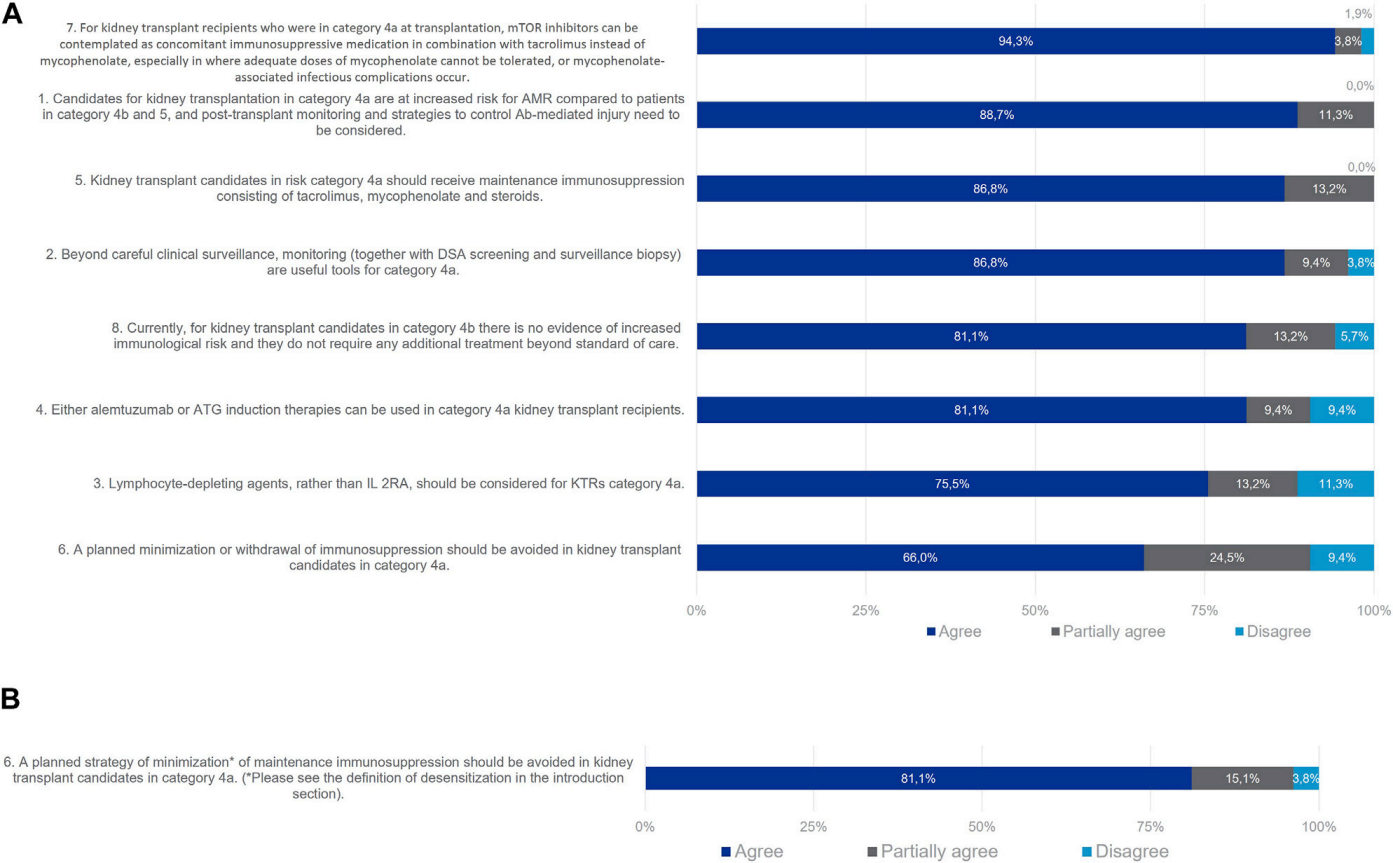
Panellist responses for Category 3 (patients with absence of DSA with but potential cellular memory against donor HLA antigens on day 0) after **(A)** Wave 1 and **(B)** wave 2.

Most members of the Delphi Review Group (89%) agreed that for Category 4a patients post-transplant monitoring and strategies to control antibody-mediated injury need to be considered (Figure 6). Useful tools beyond careful clinical surveillance are monitoring with DSA screening and surveillance biopsy (agreement rate 87%). Lymphocyte-depleting agents should be considered for patients in Category 4a, rather than IL-2Ras alone (agreement rate 76%). Alemtuzumab or ATG (i.e., T-cell depleting agents) can be used as induction therapies for this group (agreement rate 81%) since as naïve alloantibody response, recall responses also require T cell help [14]. In all, 87% of the Delphi Review Group agreed that patients in Category 4a should receive maintenance immunosuppression with tacrolimus, mycophenolate and steroids. Also, mTOR inhibitors can be contemplated in combination with tacrolimus instead of mycophenolate, especially when the latter cannot be tolerated or when infectious complications due to mycophenolate occur (agreement rate 94%). A total of 81% of the Group agreed that a planned strategy of minimization of maintenance immunosuppression should be avoided in these patients in Category 4a. Initially, no consensus was reached during Wave one, as 66% of the Group agreed, while 25% partially agreed and 9% disagreed. For Wave two, a clear definition of minimization (a planned strategy of reduction of maintenance immunosuppression consisting in reducing calcineurin inhibitor (CNI) trough levels and/or antimetabolites doses below the standard values and/or withdrawing corticosteroids) was included and consensus was achieved. However, withdrawal of steroids or lower than usual doses of tacrolimus/MMF in these patients was also considered appropriate by the Delphi Review Group, depending on time after transplantation, occurrence of acute rejection and side effects of immunosuppression.

Given the current lack of routinely accessible tests to evaluate the humoral cellular memory of kidney transplant candidates, the Delphi Review Group reached a consensus that patients in Category 4b do not necessitate additional treatment beyond the standard of care, with an agreement rate of 81%. While this finding might suggest the potential exclusion of Category 4b from the classification, the ENGAGE working group recommends retaining this category. Doing so emphasizes the critical unmet medical need and encourages research on alloreactive memory B cells. The simplification of the classification awaits robust evidence on the role of these subsets and the development of reliable assays to screen for their presence.

### Category 5 Patients (With No DSA and No Cellular Memory)

**FIGURE 7:**
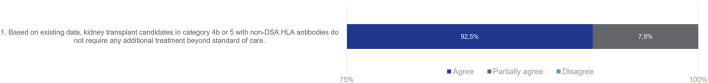
Panelist responses for category 5 (Patients with no DSA and no cellular memory on Day 0).

No specific statements were developed for this category as these transplant candidates (who present with no DSA, and no cellular memory indicating heightened risk of rejection) were not the focus of the review. It was agreed that these patients do not require any additional treatment beyond standard of care (agreement rate 93%; Figure 7).

## Discussion

Through a process of systematic literature searching, statement development and Delphi-based consensus achievement a group of European experts in the field of kidney transplantation agreed a series of recommendations for desensitization and immunomodulation strategies based on previously defined risk categories [1]. For patients in Categories 1 and 2 the recommendation is that kidney transplantation should be avoided unless no other option is available. In this situation, for patients in category 1, a CDC negative crossmatch must be obtained through desensitization before transplantation and in both categories 1 and 2, strategies to prevent and treat antibody rebound must be carefully planned. Importantly, in the survey used to establish the consensus, the focus was primarily on the combination of plasma exchange (PEX) and intravenous immunoglobulin (IVIG) for desensitization. It’s crucial to note that this choice was made for the sake of simplicity, and while historically the first therapeutic approach, it is no longer the sole option available to clinicians. Among the alternative extracorporeal therapies capable of removing circulating HLA antibodies, both double-filtration plasmapheresis (DFPP) and immunoadsorption (IA) have demonstrated efficacy for desensitization, as supported by studies [15, 16]. Currently, there is no conclusive evidence favouring one technique over another, and studies comparing different apheresis techniques for HLA desensitization are limited. For instance, a study by Maillard et al. revealed a higher relative reduction of MFI with IA compared to three consecutives daily PLEX sessions (−69% vs. −58%, respectively, *p* = 0.003), despite IA treating a lower total volume of plasma (105 ± 6 vs. 160 ± 16 mL/kg after IA and PEX, respectively) [17]. However, a significant drawback of this study was its departure from routine clinical practice, where more than one IA or three PEX sessions are typically performed. Another recent monocentric study analysing 881 sessions (107 DFPP, 54 PEX, 720 IA) in 45 patients reported successful procedures leading to HLA incompatible kidney transplantation in 39 patients (87%) after 29 (15–51) days. IA, PE, and a lower maximal DSA MFI were associated with a greater decrease in intra-session class II DSA [15]. Apart from efficacy, the choice of the apheresis technique also considers safety. Compared to PEX, IA offers semi-specific plasma treatment, eliminating the need for albumin or plasma substitution [18]. However, the rational use of fresh-frozen plasma effectively mitigates hypofibrinogenemia-induced haemorrhagic risk associated with PEX. Therefore, all three techniques exhibited good tolerance in the study by Noble et al, with severe adverse events occurring in only 1.9% of the 881 (DFPP had a slightly higher occurrence of adverse events: 6.5%; *p* < 0.01). Lastly, it’s essential to also consider the financial and practical aspects, unfortunately IA columns comes at a higher cost are not universally available across all countries [19].

For patients of category 1 to 3 other transplant options should be preferred each time possible, such as compatible living donor transplant or kidney paired donation, or awaiting on a national prioritization program if acceptable waiting times are expected according to transplant calculators that address the likelihood of a compatible deceased donor transplant for sensitized patients [20]. They require a thorough risk/benefit analysis and strategies to control antibody-mediated injury. Patients in Category 4a require post-transplant monitoring and strategies to control antibody-mediated injury as they are at increased risk for AMR compared with patients in Category 5, who do not require any additional treatment beyond standard of care [21–25].

For patients in Categories 1, 2, 3 and 4a, careful clinical surveillance and monitoring with DSA screening and surveillance biopsy is recommended [26, 27]. With regard to desensitization strategies for patients in Categories 1, 2 and 3, it is suggested that PEX and IVIg should be part of the first line treatment. Moreover, Imlifidase could be considered for deceased kidney transplantation in selected patients in Categories 1 and 2 for whom no other treatment options are available [21, 28–31]. As far as induction therapy for patients in Categories 1, 2, 3 and 4a, lymphocyte-depleting agents rather than IL-2RAs alone are recommended and either alemtuzumab or ATG can be considered [4, 21, 32, 33]. The experts also agreed that rituximab might be considered as an adjunct to prevent antibody rebound and therefore could be included at the time of transplantation as an induction agent for patients in Categories 1, 2, 3 [21, 33–36]. This approach, although not directly evaluated in the present questionnaire, is even making better sense for patients from category 4a, who have by definition lost their serological memory (disappearance of DSA from the circulation) but remain at high risk of having persisting alloreactive memory B cells [37]. In the latter, the use of rituximab (a B-cell depleting agent with a safe tolerance profile) has been suggested as an alternative to T-cell depleting agents to prevent DSA rebound without increasing the risk of infectious and cancerous complications [38].

For maintenance immunosuppression for patients in Categories 1, 2, 3 and 4a, it is recommended that they receive treatment with tacrolimus, mycophenolate and steroids. Also, in some cases mTOR inhibitors in combination with tacrolimus instead of mycophenolate can be contemplated. Planned strategies of minimization of this maintenance immunosuppression should be avoided in these patients. Immunosuppression should be adapted and maintained in these transplant recipients unless there are treatment-related adverse events that are severe enough to alter the regimen [39].

In Category 4a patients, consensus remained elusive during Wave one concerning the statement on maintenance immunosuppression. Disagreement primarily stemmed from the belief that minimizing immunosuppression should be approached on a case-by-case basis, contingent upon DSA monitoring and surveillance biopsies. For Wave two, the statement underwent a revision, incorporating a clear definition of minimization as a planned strategy involving the reduction of maintenance immunosuppression, entailing lowering CNI trough levels and/or antimetabolite doses below standard values. However, specific target levels and doses were not delineated, and/or the withdrawal of corticosteroids was suggested. Consensus was attained in Wave two, with agreement that planned minimization strategies should be avoided for these patients. Nevertheless, experts acknowledged that, based on time post-transplantation, absence of prior acute rejection history, and immunosuppression-related side effects, the consideration of steroid withdrawal or reduced tacrolimus/MMF doses could be appropriate for select patients in this category.

In regard to kidney transplantation candidates in Categories 1 and 2, no consensus emerged regarding the use of complement inhibitors. Predominant reasons for disagreement centred around the current lack of evidence supporting this recommendation, given that complement inhibition does not reduce DSA titres. While the use of complement inhibitors may not be recommended for patients with high preformed DSA titres, these agents could still prove beneficial in addressing complement-mediated injury during an episode of AMR [6, 40–42].

According to the recent guideline from a European Society of Organ Transplantation (ESOT) working group, concerning the management of kidney transplant patients with HLA antibodies [6], highly sensitized patients should be prioritized in kidney allocation schemes and linking allocation schemes may increase opportunities. If strategies for finding a compatible kidney are very unlikely to yield a transplant, desensitization may be considered balancing the benefit/risk with staying on chronic dialysis therapy for long periods of time, if not for ever, and should be preferentially performed with PLEX or immunoadsorption (IA), supplemented with IVIg and/or anti-CD20 antibody treatment. Newer therapies such as imlifidase may offer a unique opportunity, especially for deceased-donor transplant candidates, to significantly reduce, albeit only transiently, the risk for hyperacute and accelerated graft rejection and thus, may provide access to transplantation. To date, few studies have compared HLA incompatible transplantation with remaining on the waiting list, and comparisons of morbidity or quality of life do not exist. The use of Kidney-paired Exchange Programmes (KEP) is preferred to desensitization, but highly sensitized patients should not be left on a KEP list indefinitely if the option of a direct incompatible transplant exists.

To our knowledge, this is the first study undertaken as a cohesive effort to provide an international expert consensus on desensitization and immunomodulation in kidney transplant patients according to each patient recently determined humoral risk category. A high level of consensus was achieved among this group of European experts for the management of desensitization and immunomodulation strategies of kidney transplantation recipients according to defined pre-transplantation patients humoral risk profiles. The actions to be undertaken for each patient risk category may help to improve these patients’ management, access to transplantation and long-term success.
