# Supplementary material for: European Consensus on the Management of Sensitized Kidney Transplant Recipients: A Delphi Study
Source: Transpl Int. 2024 Apr 11;37:12475. doi: 10.3389/ti.2024.12475 (PMC11043529; doi:10.3389/ti.2024.12475)
Supplement: Supplementary file 1 [file DataSheet1.docx]

**European consensus on the management of sensitized kidney transplant recipients: a Delphi study**

**Supplementary material**

**Supplementary Table S1.** Literature search strategy

| **Database** | **Search dates** | **Search terms** |
| --- | --- | --- |
| Transplant Library | 01/01/1995 – 22/06/2023 | 1. HLA incompatib$.ti,ab  2. anti-HLA.ti,ab  3. HLA abs.mp  4. HLA antibod$.ti,ab  5. incompatible kidney.ti,ab  6. DSA.ti,ab  7. 22  8. or/1-7  9. limit 8 to kidney transplant |
| MEDLINE and EMBASE | 01/01/1995 – 22/06/2023 | 1. HLA incompatib$.ti,ab  2. positive crossmatch.ti,ab  3. ((kidney or renal) adj3 (transplant$ or graft$)).ti,ab  4. 1 or 2  5. 3 and 4 |
| ***Induction in sensitized kidney transplant recipients*** | | |
| Transplant Library (identification of systematic reviews) | 01/01/2000 – 10/06/2023 | 1. induction.ti,ab  2. basiliximab/  3. rituximab/  4. Thymoglobulin.mp  5. eculizumab.mp  6. Alemtuzumab/tu [Therapeutic Use]  7. IVIG.ti,ab  8. Intravenous immunoglobulin.mp  9. or/1-8  10. limit 9 to (kidney transplant and "systematic review") |
| Transplant Library (identification of randomised controlled trials) | 01/01/2000 – 10/06/2023 | 1. induction.ti,ab  2. basiliximab/  3. rituximab/  4. Thymoglobulin.mp  5. eculizumab.mp  6. Alemtuzumab/tu [Therapeutic Use]  7. IVIG.ti,ab  8. Intravenous immunoglobulin.mp  9. or/1-8  10. DSA.ti,ab  11. donor specific antibodies.mp  12. retransplant.ti,ab  13. pregnan$.ti,ab  14. CDC.mp  15. flow.mp  16. risk.ti,ab  17. or/10-16  18. 9 and 17  19. limit 18 to (exclude congress abstracts and kidney transplant and randomised controlled trial) |

ab, abstract; ti, title.

**Supplementary Table S2.** Statements for Delphi-based consensus review

| STATEMENTS for category 1 profile patients |
| --- |
| 1. Kidney transplantation should be avoided in category 1 patients unless no other option is available. |
| 1. If kidney transplantation is considered for a category 1 recipient, a CDC negative crossmatch must be obtained as the result of desensitization and before transplantation, and strategies to prevent and treat antibody (Ab) rebound must be carefully planned. |
| 1. Plasma exchange (PEX) and intravenous immunoglobulin (IVIG) should be part of the first-line desensitization strategy in category 1 kidney transplant candidates. |
| 1. Although still controversial, the use of rituximab might be considered as an adjunct to prevent Ab-mediated injury in category 1 kidney transplant candidates. |
| 1. Beyond careful clinical surveillance, monitoring with DSA screening and surveillance biopsy are useful tools for category 1 kidney transplant recipients. |
| 1. Imlifidase might be considered as a desensitization strategy for deceased kidney transplantation in very selected patients in category 1 for whom there are no other treatment options. |
| 1. Complement-inhibitors might be considered in adjunction to a desensitization strategy for kidney transplantation candidates who are risk category 1. |
| 1. Lymphocyte-depleting agents, rather than IL2RAs, should be used for kidney transplantation candidates who are in risk category 1 at induction. |
| 1. Either alemtuzumab or antithymocyte globulin (ATG) induction therapies can be used in kidney transplant candidates in risk category 1. |
| 1. Patients in risk category 1 who undergo kidney transplantation should receive maintenance immunosuppression consisting of tacrolimus, mycophenolate and steroids. |
| 1. A planned minimization or withdrawal of immunosuppression should be avoided in kidney transplant recipients who were in risk category 1 at transplantation. |
| 1. For kidney transplant recipients who were in category 1 at transplantation, mTOR inhibitors can be contemplated as concomitant immunosuppressive medication in combination with tacrolimus instead of mycophenolate, especially in cases where adequate doses of mycophenolate cannot be tolerated, or mycophenolate-associated infectious complications occur. |

| STATEMENTS for category 2 profile patients |
| --- |
| 1. Preferably, kidney transplantation should be avoided in risk category 2 patients unless no other transplant option is available. |
| 1. If kidney transplantation is considered in category 2 candidates, a flow negative crossmatch should be obtained before transplantation, and strategies to prevent and treat Ab rebound must be carefully planned. |
| 1. PEX and IVIG should be part of the first line desensitization strategy in kidney transplantation candidates in risk category 2. |
| 1. Although still controversial, the use of rituximab might be considered as an adjunct to prevent Ab-mediated injury in category 2 kidney transplant recipients. |
| 1. Beyond careful clinical surveillance, monitoring with DSA screening and surveillance biopsy are useful tools for category 2 kidney transplant recipients. |
| 1. Imlifidase might be considered as a desensitization strategy for deceased donor kidney transplantation in very selected patients in category 2 for whom there are no other treatment options. |
| 1. Complement-inhibitors might be considered in adjunction to a desensitization strategy for kidney transplantation candidates who are risk category 2. |
| 1. Lymphocyte-depleting agents, rather than IL 2RA, should be used for KTRs category 2 at induction. |
| 1. Either alemtuzumab or ATG induction therapies can be used in category 2. |
| 1. Patients in risk category 2 who undergo kidney transplantation should receive maintenance immunosuppression consisting of tacrolimus, mycophenolate and steroids. |
| 1. A planned minimization or withdrawal of immunosuppression should be avoided in kidney transplant recipients in risk category 2 at transplantation. |
| 1. For kidney transplant recipients who were in category 2 at transplantation, mTOR inhibitors can be contemplated as concomitant immunosuppressive medication in combination with tacrolimus instead of mycophenolate, especially in where adequate doses of mycophenolate cannot be tolerated, or mycophenolate-associated infectious complications occur. |

| STATEMENTS for category 3 profile patients |
| --- |
| 1. Other transplant options (e.g., compatible living donor transplant, kidney paired donation) should be considered for kidney transplant recipients in category 3. |
| 1. Kidney transplantation in category 3 candidates requires a thorough risk/benefit analysis, and strategies to prevent and treat Ab rebound need to be carefully planned. |
| 1. PEX and IVIG might be part of a desensitization strategy, if required, in kidney transplant candidates in category 3. |
| 1. Although still controversial, the use of rituximab might be considered as an adjunct to prevent Ab-mediated injury in kidney transplant candidates in category 3. |
| 1. Beyond careful clinical surveillance, monitoring with DSA screening, surveillance biopsy are useful tools for category 3. |
| 1. Lymphocyte-depleting agents, rather than IL 2RA, should be used for KTRs category 3. |
| 1. Either alemtuzumab or ATG induction therapies can be used in category 3. |
| 1. Candidates for transplantation in risk category 3 who receive a kidney should receive maintenance immunosuppression consisting of tacrolimus, mycophenolate and steroids. |
| 1. A planned minimization or withdrawal of immunosuppression should be avoided in kidney transplant recipients in category 3. |
| 1. For kidney transplant recipients who were in category 3 at transplantation, mTOR inhibitors can be contemplated as concomitant immunosuppressive medication in combination with tacrolimus instead of mycophenolate, especially in where adequate doses of mycophenolate cannot be tolerated, or mycophenolate-associated infectious complications occur. |

| STATEMENTS for category 4 profile patients |
| --- |
| 1. Candidates for kidney transplantation in category 4a are at increased risk for AMR compared to patients in category 4b and 5, and post-transplant monitoring and strategies to control Ab-mediated injury need to be considered. |
| 1. Beyond careful clinical surveillance, monitoring (together with DSA screening and surveillance biopsy) are useful tools for category 4a. |
| 1. Lymphocyte-depleting agents, rather than IL 2RA, should be considered for KTRs category 4a. |
| 1. Either alemtuzumab or ATG induction therapies can be used in category 4a kidney transplant recipients. |
| 1. Kidney transplant candidates in risk category 4a should receive maintenance immunosuppression consisting of tacrolimus, mycophenolate and steroids. |
| 1. A planned minimization or withdrawal of immunosuppression should be avoided in kidney transplant candidates in category 4a. |
| 1. For kidney transplant recipients who were in category 4a at transplantation, mTOR inhibitors can be contemplated as concomitant immunosuppressive medication in combination with tacrolimus instead of mycophenolate, especially in where adequate doses of mycophenolate cannot be tolerated, or mycophenolate-associated infectious complications occur. |
| 1. Currently, for kidney transplant candidates in category 4b there is no evidence of increased immunological risk and they do not require any additional treatment beyond standard of care. |

| STATEMENTS for category 5 profile patients |
| --- |
| 1. Based on existing data, kidney transplant candidates in category 4b or 5 with non-DSA HLA antibodies do not require any additional treatment beyond standard of care. |

**Supplementary Table S3.** The Delphi Review Group selection

| **Strategy** | **Experts recruited**  **N=53** |
| --- | --- |
| 1. **Open call sent by ESOT to its member list with the following questions:** 2. How many years of experience do you have in the care of kidney transplantation recipients? 3. <5 4. 6-10 5. 11-20 6. >20 7. Do you have experience in desensitization procedures in sensitized kidney transplantation recipients? 8. Yes, < 5 cases 9. Yes, 6-20 cases 10. Yes, >20 cases 11. No 12. Do you have experience in immunosuppressive therapy in sensitized kidney transplantation recipients? 13. Yes, < 5 cases 14. Yes, 6-20 cases 15. Yes, >20 cases 16. No 17. Have you contributed to scientific publications in the field of sensitized kidney transplant recipients? 18. Yes (please provide reference) 19. No 20. Have you participated as PI or co-PI to a clinical trial aimed to develop strategies of desensitization/immunosuppression and to evaluate the outcome of kidney transplantation in highly sensitized recipients? 21. Yes 22. No | 12 |
| 1. **Call for participation Kidney specific sections of the scientific societies (ESOT and ERA-EDTA).** | 23 |
| 1. **Call for participation to members of 3 previous working groups on this topic (TLJ WS06, Engage 1 and Engage 2).** | 18 |

**Supplementary Table S4.** The Delphi Review Group

| **Characteristic** | **Delphi Review Group**  **N=53** |
| --- | --- |
| Geographical location, n  United Kingdom  France  Italy  Spain  Belgium  The Netherlands  Germany  Austria  Switzerland  Norway  Sweden  Denmark  Hungary  The Czech Republic  Romania  Portugal  Turkey | 10  10  8  5  3  3  2  2  2  1  1  1  1  1  1  1  1 |
| Mean age, years (range) | 50.5 (36–67) |
| Male sex, % | 72 |
| Specialty, %  Nephrologists  Transplant surgeons  Immunologists | 71.7  24.5  3.8 |
| Academic status, %  Full professor  Associate professor  Assistant professor  Other | 38  19  9  34 |
| Practice based in a University Hospital, % | 93 |
| Practice based in a public centre, % | 89 |
| >5 years’ experience in the care of kidney transplant recipients, % | 100 |
| Undertaken desensitization procedures for kidney transplantation, %  ≥6 patients  ≥20 patients | 15  64 |
| Overseen the management of immunosuppressive therapy in sensitized kidney transplant recipients, %  ≥6 patients  ≥20 patients | 21  72 |
| Contributed to scientific publications in the field of sensitized kidney transplant recipients, % | 72 |
| Participated as a primary or co-primary investigator in a clinical trial aimed at developing strategies of desensitization/immunosuppression and evaluating the outcome of kidney transplantation in sensitized recipients, % | 45 |

**Supplementary Figure S1:** ENGAGE II review and question preparation processes.

January 2021

Planning of the activities and selection/involvement of the Working Group Members

February/March

Definition of the relevant PICO’s by the Expert Working SubGroups

April

Systematic searches by the Centre for Evidence in Transplantation

June

Plenary discussion with all the Subgroups member and preparation of statements

July

First draft of the White paper, including statements and

explanatory notes and rationale specific to each statement

October

Circulation of the draft, Incorporation of reviewer comments

November

Expert Working Group meeting to finalise text and statements

December

White paper delivered to ESOT, and available for the development of the Delphi Consensus activity
